# A sensitive and simple RT-LAMP assay for sarbecovirus screening in bats

**DOI:** 10.1128/spectrum.02591-23

**Published:** 2023-11-16

**Authors:** Tony Tat-Yin Chan, Franklin Wang-Ngai Chow, Joshua Fung, Flora Ka-Kei Cheng, George Chi-Shing Lo, Chi-Ching Tsang, Hayes Kam-Hei Luk, Antonio Cheuk-Pui Wong, Zirong He, Kam Leng Aw-Yong, Xueyan Liu, Kwok-Yung Yuen, Patrick Chiu-Yat Woo, Susanna Kar-Pui Lau

**Affiliations:** 1 Department of Microbiology, Li Ka Shing Faculty of Medicine, The University of Hong Kong, Hong Kong, China; 2 Department of Health Technology and Informatics, The Hong Kong Polytechnic University, Hong Kong, China; 3 School of Medical and Health Sciences, Tung Wah College, Hong Kong, China; 4 Doctoral Program in Translational Medicine and Department of Life Sciences, National Chung Hsing University, Taichung, Taiwan; 5 The iEGG and Animal Biotechnology Research Center, National Chung Hsing University, Taichung, Taiwan; University of Prince Edward Island, Charlottetown, Prince Edward Island, Canada

**Keywords:** RT-LAMP, sarbecovirus, bat screening, coronavirus, SYTO9, molecular detection

## Abstract

The availability of simple, inexpensive assays for coronavirus (CoV) detection is critical for conducting animal surveillance studies to prevent new zoonotic epidemics. We have previously developed a colorimetric reverse transcription loop-mediated isothermal amplification (RT-LAMP) assay for diagnosing coronavirus disease 2019 (COVID-19) in humans using respiratory samples. In this study, we improved and extended the application of this assay to detect other sarbecoviruses in bats. Twenty-four and twenty-one out of 838 oral and alimentary samples collected during 2017–2019 were sarbecovirus-positive by quantitative reverse transcription PCR (qRT-PCR) and our colorimetric RT-LAMP assay, respectively. PCR and Sanger sequencing of the partial spike (S) gene in the S1 subunit region and RNA-dependent RNA polymerase gene of the 21 sarbecovirus-positive samples showed that they were all closely related to bat SARS-related coronavirus HKU3. A green fluorescent nucleic acid stain, SYTO9, was also added for real-time quantification and evaluated in the colorimetric RT-LAMP assay. We observed a positive correlation (Spearman’s rank correlation coefficient of 0.77, *P* < 0.0001) between the time to positivity in the colorimetric RT-LAMP assay and cycle threshold (Ct) values in qRT-PCR assay, suggesting that our assay allows quantitative analysis of viral load in samples. This easily performed, highly sensitive, and specific colorimetric RT-LAMP assay could facilitate mass screening for sarbecoviruses in bats and other animal populations. It will be particularly useful for field studies without sophisticated laboratory equipment and expertise.

**IMPORTANCE:**

We report the application of a colorimetric and fluorescent reverse transcription loop-mediated isothermal amplification (RT-LAMP) assay to facilitate mass screening for sarbecoviruses in bats. The assay was evaluated using a total of 838 oral and alimentary samples from bats and demonstrated comparable sensitivity and specificity to quantitative reverse transcription PCR (qRT-PCR), with a simple setup. The addition of SYTO9, a fluorescent nucleic acid stain, also allows for quantitative analysis. The scalability and simplicity of the assay are believed to contribute to improving preparedness for detecting emerging coronaviruses by applying it to field studies and surveillance.

## INTRODUCTION

The coronavirus disease 2019 (COVID-19) pandemic has highlighted the urgent need for rapid diagnosis of severe acute respiratory syndrome coronavirus 2 (SARS-CoV-2) infections in order to contain the outbreak in its early stages to reduce the burden of disease (1, 2). Despite its high sensitivity and reliability, quantitative reverse transcription PCR (qRT-PCR), the gold standard for nucleic acid diagnosis (3), has a relatively long turn-around time. It also requires expensive instruments and experienced laboratory technicians, and it lacks portability. As a result, it has not met the huge demand generated during the pandemic. To address the strong demand for rapid COVID-19 diagnosis, we have previously developed a rapid, simple, and inexpensive colorimetric COVID-19 loop-mediated isothermal amplification (LAMP) in early 2020 (4). Briefly, the reverse transcription loop-mediated isothermal amplification (RT-LAMP) assay combines three pairs of degenerate primers (outer, inner, and loop primers) targeting the envelope (E) gene of sarbecoviruses, reverse transcriptase, DNA polymerase, and a pH indicator. During DNA polymerization, nucleotides are added to the growing DNA strand, which releases a hydrogen ion (H^+^) in the process and causes a decrease in the pH of the solution, resulting in a color change from pink to yellow. The RT-LAMP assay only requires incubation in a heat block for 60 to 90 minutes, depending on the viral RNA copy number, without the need for a thermal cycler or a quantitative PCR (qPCR) machine.

Bats are reservoirs of a large variety of zoonotic viruses (5, 6), including Marburg virus, Nipah virus, Hendra virus, and coronaviruses (CoVs) (7 – 9). In particular, a large diversity of CoVs, including SARS-related CoVs (SARSr-CoVs), have been consistently found to be circulating in bats (5, 10, 11). Some of these circulating CoVs have a high capacity for jumping the species barrier and pose a constant threat to human populations (6). For instance, bat coronaviruses closely related to SARS-CoV and SARS-CoV-2 were previously detected, all of which belonged to the CoV subgenus *Sarbecovirus*. While strong evidence suggests bats being the natural reservoir of SARS-CoV (12 – 14), the exact origin of SARS-CoV-2 remains to be ascertained, as only part of the genome of SARS-CoV-2 was shown to be closely related to bat and pangolin viruses (9, 15, 16). In order to detect the future emergence of similar or novel bat CoVs, continuous animal surveillance is essential (17, 18). However, the predominant screening method currently in use, RT-PCR, has a long turn-around time, takes up significant manpower, and requires expensive equipment, which significantly limits its efficiency and practicality in resource-limited settings.

**Fig 1:**
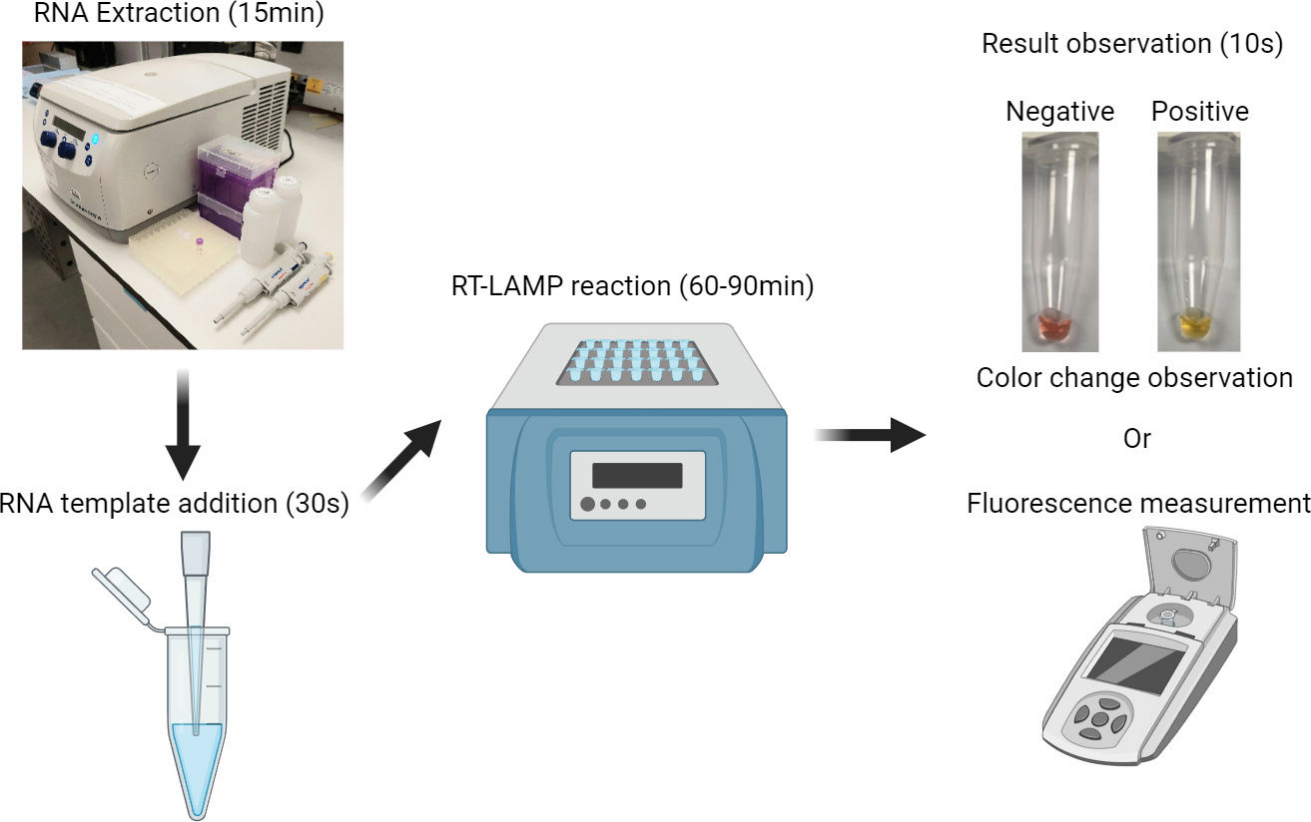
Illustration of the workflow of RT-LAMP assay for sarbecoviruses screening in bats.

In this work, we further explored the potential of RT-LAMP assay for mass animal screening. We tested our colorimetric RT-LAMP assay for the detection of sarbecoviruses in bats (order *Chiroptera*) using oral and alimentary swab samples collected in Hong Kong, China, as shown in Fig. 1. The positive RT-LAMP products were then confirmed by Sanger sequencing with LAMP partial inner primers. qRT-PCR using the N3 primers and probe set of the CDC 2019 nCoV Real-Time RT-PCR Diagnostic Panel was used for further sarbecovirus confirmation and RT-LAMP assay comparison. To allow for quantitative analysis, a green fluorescent nucleic acid stain, SYTO9, was also included and evaluated in the RT-LAMP assay. In addition, we performed Sanger sequencing and phylogenetic analyses of the partial spike (S) and RNA-dependent RNA polymerase (RdRp) regions in 21 bat SARS-like CoVs identified in this study.

## MATERIALS AND METHODS

### Bat samples

A total of 838 bat oral and alimentary swab samples (419 samples each) were collected by the Agriculture, Fisheries and Conservation Department (AFCD), the Government of the Hong Kong Special Administrative Region (HKSAR), China, during the period of 2017 to 2019 from different locations in Hong Kong using previously described procedures (5). In brief, the swab samples were collected by field biologists using sterile disposable swabs with full personal protective equipment, and biosafety practices were employed during sample collection. To prevent cross-contamination, protective gloves were changed between handling different samples. Immediately after collection, all swab samples were placed in a viral transport medium and transported to the laboratory. They were stored at 4°C prior to processing. The collection of bat samples has been approved by the AFCD of the HKSAR and the Committee on the Use of Live Animals in Teaching and Research, The University of Hong Kong (CULATR Ref. No.: 2284-10 and 3330-14, respectively; date of approval: 23 March 2011 and 17 April 2014, respectively).

### RNA extraction

The swab samples suspended in the viral transport medium were subjected to viral RNA extraction using the QIAamp Viral RNA Mini kit (Qiagen, Germany) following the manufacturer’s instructions. In brief, 140 µL of swab samples in the viral transport medium was inactivated using buffer AVL within a biosafety level 2 cabinet and subsequently used for extraction. The RNA was then eluted using 60 µL of buffer AVE. The extracted RNA and remaining swab samples were both stored at −80°C upon usage.

### Detection of sarbecoviruses by RT-LAMP assay and qRT-PCR

The extracted RNAs from bat samples were subjected to RT-LAMP assay under the optimized condition as described previously (4) with further modifications. RNAs extracted from SARS-CoV and SAR-CoV-2 cultures were used as positive controls, whereas RNAs extracted from *Tylonycteris* bat coronavirus HKU4 (subgenus *Merbecovirus*), Middle East respiratory syndrome coronavirus (MERS-CoV; subgenus *Merbecovirus*), human coronavirus HKU1 (subgenus *Embecovirus*), human coronavirus OC43 (subgenus *Embecovirus*), and other human CoV samples were used as negative controls. In brief, the optimized 25-µL colorimetric RT-LAMP reaction consisting of 12.5 µL of WarmStart Colorimetric LAMP 2 × Master Mix (New England Biolabs, MA, USA), 7.5 µL of primer mixture [outer primer (F3, B3: 1.36 µM), inner primer (FIP, BIP: 5.43 µM), and loop primer (LoopF, LoopB: 2.73 µM)], and 5 µL of RNA template was incubated at 60°C for 90 minutes in a heat block. A color change from pink to yellow or amber was interpreted as positive, while pink or coral pink color was regarded as negative. The positive samples were confirmed by three additional technical replicates. The positive RT-LAMP products were further verified by Sanger sequencing using F2 and B2 primers.

The N3 primers and probe set of the CDC 2019 nCoV Real-Time RT-PCR Diagnostic Panel (IDT, Coralville, IA, USA) and SuperScript III Platinum One-Step qRT-PCR Kit (Thermo Fisher Scientific, MA, USA) were also used to examine bat samples. In brief, each 20-µL reaction mixture consisted of 10 µL of 2 × SuperScript III Platinum Master Mix, 1.5 µL of SARS-CoV-2 CDC qPCR N3 primers and probe mix, 1 µL of SuperScript III RT/Platinum Taq Mix, and 5 µL of RNA template. The one-step qRT-PCR condition includes reverse transcription for 15 minutes at 50°C, pre-denaturation for 2 minutes at 95°C, 45 cycles of denaturation for 3 s at 95°C, and annealing and elongation for 30 s at 55°C, with fluorescent detection at the end of each cycle.

### RT-LAMP assay evaluation with the addition of SYTO9

To allow for quantitative analysis of the colorimetric RT-LAMP assay, a green fluorescent nucleic acid stain, SYTO9 (Invitrogen, CA, USA), was added to the RT-LAMP assay. In brief, 5 µL of RNA template were used in RT-LAMP reactions together with SYTO nine at a final concentration of 2 µM. Fluorescence readings were taken over a 2-hour period at a 2-minute interval with the LightCycler 480 Real-Time PCR System (Roche, Switzerland). Spearman’s correlation was performed to study the correlation between time to positivity by colorimetric RT-LAMP assay with SYTO9 and CDC N3 probe assay cycle threshold (Ct) values by using the GraphPad Prism version 8.14 (GraphPad Software, San Diego, CA, USA).

### RT-PCR and DNA sequencing of the S and RdRp regions

Fifty-two sarbecovirus genomes that are available at the National Center of Biotechnology Information (NCBI) GenBank database were retrieved and aligned. Two pairs of primers targeting S1 (5′- AATGTHRTHAGHGGYTGGRTYTTYGG-3′ and 5′- TGTATGGTARCTAGCACAAATGCCWG-3′) and RdRp regions (5′- GGTTGGGAYTAYCCAAARTGTGA-3′ and 5′- GGCRTCRTCAGAAAGAATCATCA-3′) of the aligned sarbecovirus genomes were designed separately for RT-PCR, followed by Sanger sequencing. Extracted RNAs from bat samples positive in our RT-LAMP assay were subjected to RT-PCR. The amplified PCR products were evaluated by gel electrophoresis. The distinct bands were gel purified with the QIAquick Gel Extraction Kit (Qiagen, Germany) and were sequenced with an ABI Prism 3130 × genetic analyzer (Applied Biosystems, MA, USA).

### Phylogenetic analysis

The partial S1 and RdRp sequences were aligned and compared with other sarbecoviruses available from GenBank using the server MAFFT version 7 (19). Smart Model Selection from PhyML was first used to calculate the best-fitting substitution model for maximum-likelihood analyses. Partial S1 and RdRp gene trees were reconstructed using the substitution model GTR + G + I and T92 + G, respectively. Maximum-likelihood phylogenetic trees with 1,000 bootstrap replicates of partial S1 and RdRp were then reconstructed using Molecular Evolutionary Genetics Analysis version 10.0 (MEGA X) (20).

## RESULTS

### Detection of sarbecoviruses by RT-LAMP assay and qRT-PCR

A total of 838 oral and alimentary samples were collected from 14 distinct bat species in various locations in Hong Kong, China. Among these samples, 24 alimentary samples (2.86%) were identified as positive for sarbecovirus using qRT-PCR, and 21 of such alimentary samples (2.51%) were also detected as sarbecovirus-positive in the colorimetric RT-LAMP assay as displayed in Table 1. This indicates a sensitivity of 87.50% using the results from qRT-PCR as a comparison (95% CI: 0.87–0.88). The 21 samples that tested positive in the RT-LAMP assay were subsequently confirmed using Sanger sequencing of the RT-LAMP products with LAMP partial inner primers. All positive samples were from Chinese horseshoe bats (*Rhinolophus sinicus*). None of the 14 RNA samples with other coronaviruses not belonging to *Sarbecovirus* exhibited positive results in the RT-LAMP at 90 minutes, demonstrating 100% specificity.

**TABLE 1:** Detection of CoV in different bat species by RT-LAMP and qRT-PCR^*a*^

Scientific name	Common name	No. of bats captured	No. of bats positive for CoV by RT-LAMP (%)	No. of bats positive for CoV by qRT-PCR (%)	Sensitivity (95% CI)	CoV detected^*b*^	Sampling location of bats
*Hipposideros armiger*	Great roundleaf bat	29	0	0		–	SK
*Hipposideros pomona*	Pomona leaf-nosed bat	145	0	0		–	TLC
*Miniopterus magnater*	Western bent-winged bat	84	0	0		–	LT, SK, TLC
*Miniopterus pusillus*	Small bent-winged bat	67	0	0		–	FY, SK, TLC
*Miniopterus* sp.	Bent-winged bat	39	0	0		–	SK
*Myotis chinensis*	Large myotis	19	0	0		–	SK
*Myotis horsfieldii*	Horsfield’s bat	7	0	0		–	SK
*Myotis ricketti*	Rickett’s big-footed bat	40	0	0		–	LMH, SK, TLC
*Nyctalus plancyi*	The Chinese noctule	1	0	0		–	YSO
*Pipistrellus abramus*	Japanese pipistrelle	1	0	0		–	YSO
*Rhinolophus affinis*	Intermediate horseshoe bat	140	0	0		–	FY, LT, SK, TLC
*Rhinolophus pusillus*	Least horseshoe bat	11	0	0		–	SK, TLC
*Rhinolophus sinicus*	Chinese horseshoe bat	223	21 (9.42)	24 (10.8)	87.50% (0.87–0.88)	Bat CoV HKU3	FY, LMH, LT, SK, TLC
*Tylonycteris pachypus*	Lesser bamboo bat	32	0	0		–	TLC

^*a*^ FY, Fung Yuen; LMH, Lin Ma Hang Lead Mine; LT, Lantau Island; SK, Sai Kung; TLC, Tai Lam Chung; YSO, Yung Shue O Stream.

^*b*^ –, undetected.

### RT-LAMP assay evaluation with the addition of SYTO9

**Fig 2:**
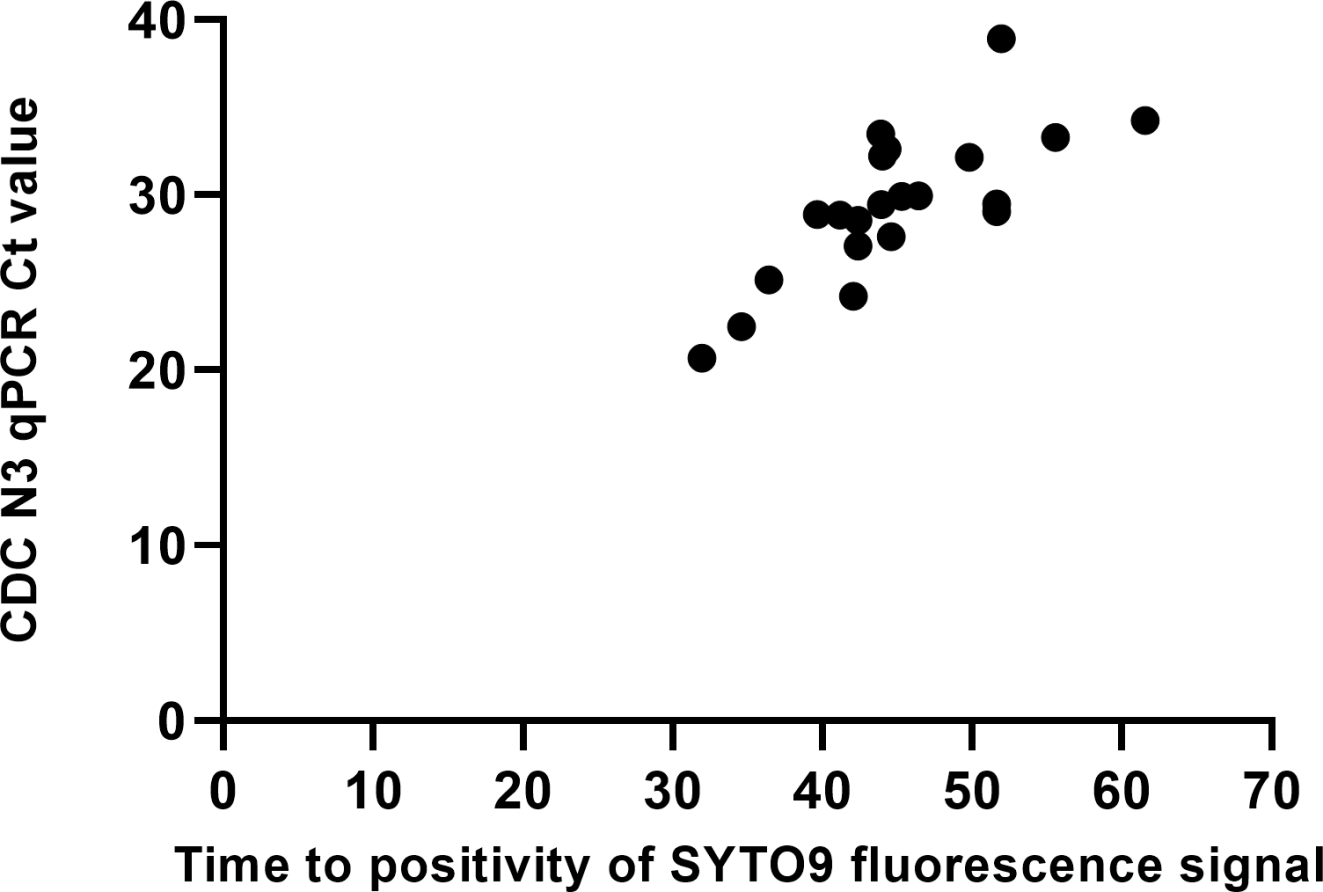
Relationship between CDC N3 qPCR Ct value and time to positivity of SYTO9 fluorescence signal.

The colorimetric RT-LAMP assay was also evaluated together with the addition of SYTO9, a green fluorescent nucleic acid stain. All 21 RT-LAMP tested positive samples were able to show positive color change and fluorescence reading as shown in Table 2, indicating that our colorimetric RT-LAMP assay is compatible with the addition of SYTO9. With the addition of SYTO9, a viral titer of each sample could be estimated. There was a positive correlation between the time to positivity in colorimetric RT-LAMP assay with SYTO9 and CDC N3 probe assay Ct values, with a Spearman’s rank order correlation coefficient of 0.77 (*P* < 0.0001) as shown in Fig. 2. Samples with higher viral loads turned positive at an earlier time. Therefore, the addition of SYTO9 in our colorimetric RT-LAMP assay allowed the quantitative analysis of the viral titer in a sample without affecting the performance and readout of the assay.

**TABLE 2:** RT-LAMP assay evaluation with the addition of SYTO9^*a*^

Samples	SYTO9 fluorescence reading (minutes for positive results)	CDC N3 probe assay qRT-PCR (Ct value)	Colorimetric RT-LAMP result observation
SK37A21	34.60	22.49	+
SK55A21	31.96	20.69	+
TLC12A9	51.64	29.49	+
TLC46A9	44.60	27.61	+
TLC47A9	55.56	33.29	+
TLC65A9	42.38	28.54	+
TLC68A9	61.56	34.26	+
TLC70A9	36.42	25.14	+
TLC3A7	39.66	28.89	+
TLC21A7	41.16	28.84	+
TLC37A7	44.02	32.21	+
TLC28A21	45.30	29.90	+
TLC29A21	44.32	32.62	+
TLC19A	46.42	29.96	+
TLC22A	51.64	29.06	+
TLC23A	43.92	29.46	+
TLC31A	51.92	38.93	+
TLC42A	43.88	33.49	+
TLC53A	49.80	32.14	+
TLC60A	42.38	27.07	+
TLC74A	42.06	24.20	+

^*a*^ +, positive; −, negative.

### Phylogenetic analyses

**Fig 3:**
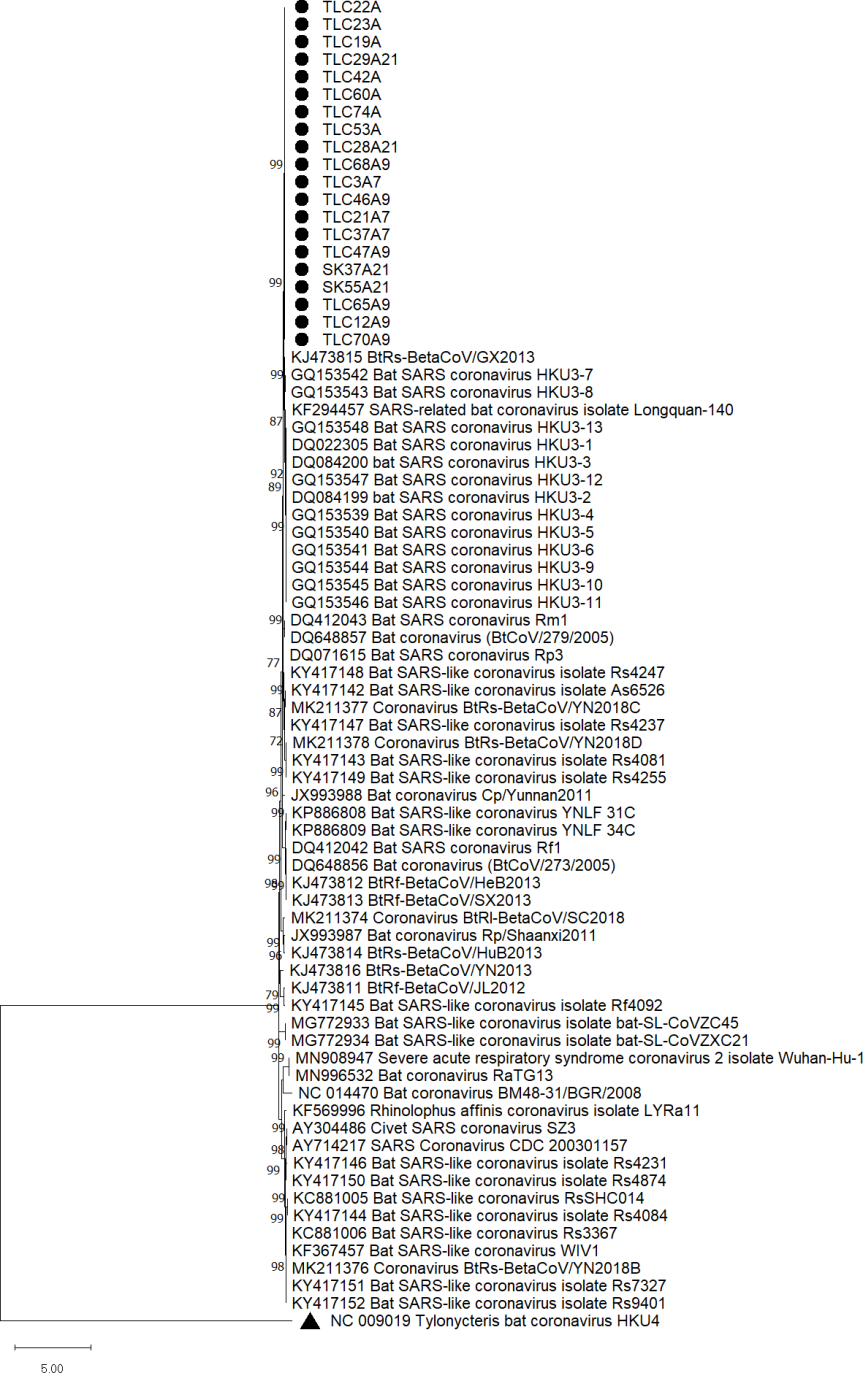
Phylogenetic analysis of partial S1 fragment (1,623 bp) nucleotide sequences of the 20 S1-positive samples with other sarbecoviruses and *Tylonycteris* bat coronavirus HKU4 as an outgroup. The partial S1 gene tree was reconstructed by the maximum likelihood method using the substitution model GTR + G + I. The bootstrap values were calculated from 1,000 trees. Only bootstrap values >70% are labeled. The scale bar represents the number of substitutions per site. The screened 20 samples are marked with black dots. *Tylonycteris* bat coronavirus HKU4 is marked with a black triangle.

**Fig 4:**
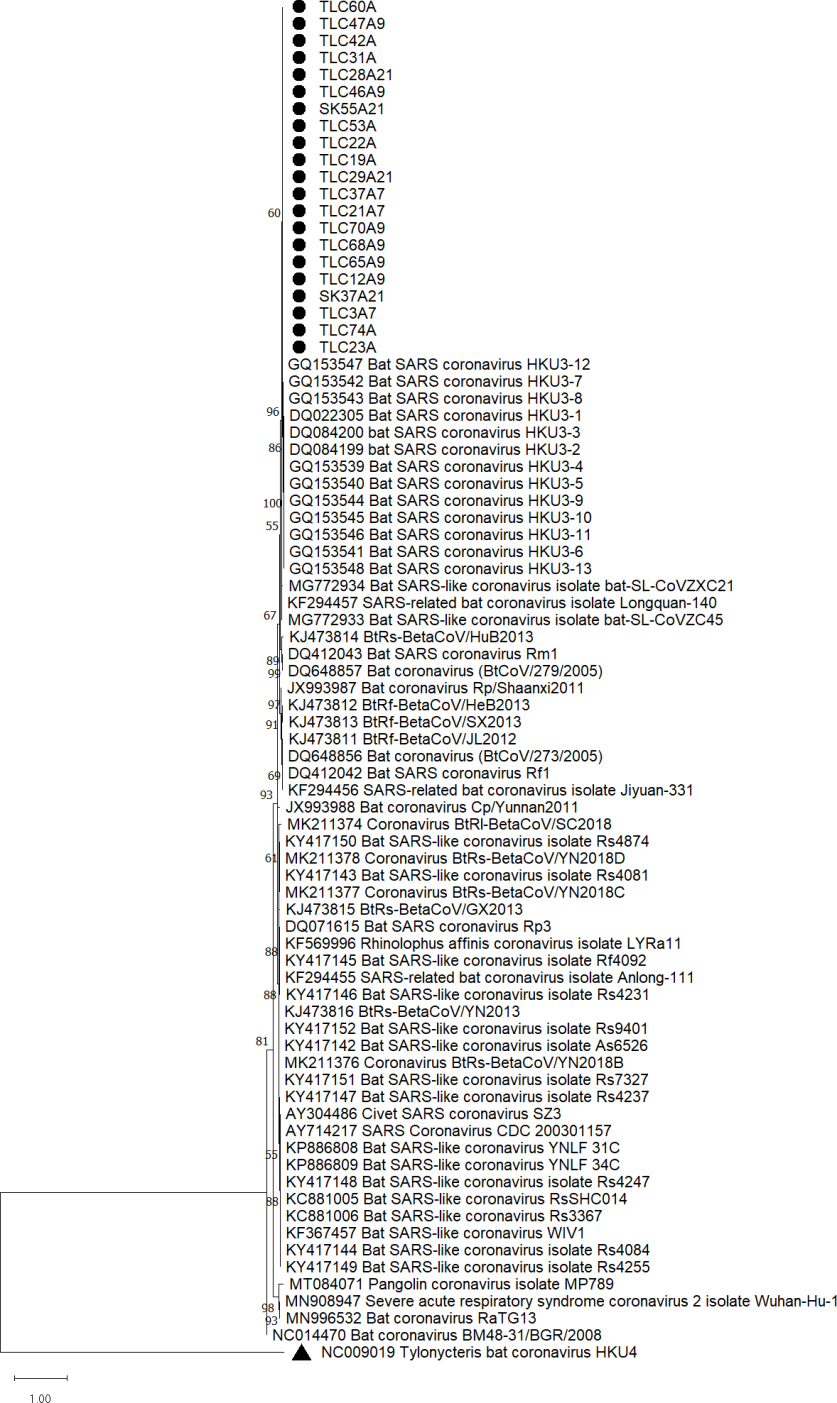
Phylogenetic analysis of partial RdRp fragment (440 bp) nucleotide sequences of the 21 positive samples with other sarbecoviruses and *Tylonycteris* bat coronavirus HKU4 as an outgroup. The partial RdRp gene tree was reconstructed by the maximum likelihood method using the substitution model T92 + G. The bootstrap values were calculated from 1,000 trees. Only bootstrap values >70% are labeled. The scale bar represents the number of substitutions per site. The screened 21 samples are marked with black dots. *Tylonycteris* bat coronavirus HKU4 is marked with a black triangle.

After confirming the presence of sarbecoviruses in these 21 samples, phylogenetic trees were reconstructed using the nucleotide sequences of S1 and RdRp as shown in Fig. 3 and 4. All samples were positive in RT-PCR using RdRp primers. However, RT-PCR with S1 primers failed to detect and sequence one of the samples (TLC31A), possibly due to a low viral load as shown in qRT-PCR. The RT-LAMP positive samples formed a distinct cluster in both phylogenetic trees inferred from the S and RdRp sequences, being more closely related to bat SARS coronavirus HKU3 than other sarbecoviruses. Phylogenetic analyses also revealed that the positive samples were more closely related to bat SARSr-CoVs and SARS-CoV than to SARS-CoV-2.

## DISCUSSION

In this study, we extended the application of our previously developed colorimetric RT-LAMP assay to detect other sarbecoviruses in bats and demonstrated that the assay is sensitive and specific for their detection. Our RT-LAMP assay successfully detected sarbecoviruses from 21 bat alimentary swab samples with Ct values ranging from 20.69 to 38.93, as determined with qRT-PCR using the CDC N3 probe assay. The samples achieved in our laboratory, previously confirmed positive for other subgenera of CoV under *Betacoronavirus*, including *Merbecovirus* and *Embecovirus,* were also subjected to the RT-LAMP assay. No false positives were observed, indicating the assay is highly specific. Although it is possible that our current assay may yield false positives due to the inclusion of degenerate bases in the primers and prolonged reaction times, we have taken steps to optimize the assay conditions, such as adjusting the concentration of inner primers, to ensure high specificity and minimize the likelihood of false-positive results.

Furthermore, advancements were also made on our previous COVID-19 LAMP assay, as shown in Table 3. In the previous version, the reading of the color change may have been challenging or biased, so we propose the addition of an extra detection dye to our assay. There is an increasing number of LAMP assays that use intercalating fluorescence dyes such as SYBR Green, EvaGreen, and SYTO9, which bind to double-stranded DNA, allowing real-time data collection (21 – 24). Therefore, in addition to phenol red, a pH indicator originally presented in our colorimetric RT-LAMP assay, we also evaluated the addition of a green fluorescent nucleic acid stain, SYTO9. It was shown that the addition of SYTO9 is compatible with our colorimetric RT-LAMP assay, with no contradictions being observed in color change and fluorescence reading readouts. The fluorescence measurement can be achieved using a simple portable fluorometer, which can cost as low as approximately USD 60, or a commercial one with a cost of approximately USD 1,500 (25). Moreover, in order to perform real-time result reading and quantitative analysis of the viral titers of the samples by comparing time to positivity, the fluorescence can be measured in real time by placing the fluorometer atop the RT-LAMP assay heat block by enclosing in a compact 3D printed housing (26). Alternatively, there is also a newly developed molecular detection system, eGGi, specifically designed for on-site testing based on the isothermal amplification method (27). Our colorimetric RT-LAMP assay offers great flexibility, as different indicators could be added to our colorimetric RT-LAMP assay according to individual needs and preferences after evaluation.

**TABLE 3:** Comparison of RT-LAMP with COVID-19 LAMP, RT-PCR, qRT-PCR, and metagenomic technologies^*a*^

	RT-LAMP (this study)	COVID-19 LAMP (4)	RT-PCR	qRT-PCR	iSeq 100 Sequencing System^*a*^
Assay time	30–90 minutes	30–90 minutes	>4 hour	90 minutes	9–18 hour
Users	Junior lab technologists	Junior lab technologists	Junior lab technologists	Experienced lab technologists	Experienced lab technologists
Costs/test (USD)	$2–7	$2–4	$4–8	$20–60	$25–150
Machine cost (USD)	$160–1500	$100–300	>$9,000	>$45,000	$19,900
Apparatus	Heat block; fluorometer (SYTO-9 version)	Heat block	Thermal cycler	qRT-PCR machine	iSeq 100 Sequencing System
Capacity	Unrestricted (up to 48 samples per block)	Unrestricted (up to 48 samples per block)	96 samples per run	96/384 samples per run	One sample per run
Results readout	Easy—visual inspection or fluorometer reading	Easy—visual inspection only	Difficult—an extra step of gel electrophoresis	Not easy—Ct calculations	Easy—direct report by the machine
Quantitative analysis	Possible	No	No	Yes	Yes
Feasibility for field study	Feasible	Feasible	Not feasible	Not feasible	Not feasible

^*a*^ ISeq100 is chosen for comparison here as it is a commonly used system worldwide. It is a compact benchtop NGS system developed by Illumina, which is designed for small whole-genome sequencing (e.g., bacteria, viruses, and plasmids) by targeting a set of genes or gene regions, gene expression analysis, or 16S metagenomics.

This colorimetric RT-LAMP assay can facilitate rapid and massive surveillance of sarbecoviruses in bats in future coronavirus epidemics of zoonotic origin. So far, most studies involving the detection of bat CoVs have relied on RT-PCR, qRT-PCR, or metagenomic technologies (28 – 30). Despite the advantage of full genome analysis, metagenomics using next-generation sequencing (NGS) has a relatively long turnaround time when compared to RT-LAMP, RT-PCR, and qRT-PCR, as summarized in Table 3. Our RT-LAMP assay not only has a significantly shorter assay time compared to RT-PCR and NGS workflow, but it is also comparable or even faster to that of qRT-PCR. Our RT-LAMP assay does not require expertise in molecular technology, bioinformatics, or expensive machinery, which could be challenging for laboratories in developing countries. In addition to having a simple experimental setup, the result readout of our RT-LAMP assay is also easy and convenient, requiring only visual inspection or fluorescence measurement with a simple fluorometer. Moreover, it can even allow quantitative analysis in such a simple setup by the addition of SYTO9, which only costs around USD 3 per reaction. Furthermore, the cost-per-test of our RT-LAMP assay is the most economical among the four types of nucleic acid-based diagnostic assays, highlighting its potential to be scaled up for mass surveillance with comparable sensitivity to RT-PCR and qRT-PCR. We have illustrated that the sensitivity of our RT-LAMP assay is comparable to qRT-PCR in CoVs screening of bat samples. Additionally, samples without prior RNA extraction can also be subjected to RT-LAMP assay (31), by adding direct samples to the lysis buffer (buffer TE, Tween 20, thermolabile proteinase K, and DNase) with the RT-LAMP reaction mixture. The entire process can be conducted without a centrifuge and extraction. Therefore, with further development, our RT-LAMP assay, which only requires simple equipment requirement, could be adapted for field studies without the need for RNA extraction. The application of this straightforward, economical, highly sensitive, and specific colorimetric RT-LAMP assay will facilitate bat surveillance to monitor sarbecoviruses in bat and related animal populations such as ferrets, hamsters, macaques, mice, monkeys, rabbits, and tree shrews, which are susceptible to SARS-CoV-2 in the laboratory setting (32, 33).

Our results also support that horseshoe bats (*Rhinolophus* spp.) should remain the major target for sarbecoviruses screening. Positive samples identified in this study were all from Chinese horseshoe bats, indicating it as the major bat species serving as the sarbecoviruses reservoir in Hong Kong. This finding is also in line with other previous studies that sarbecoviruses are commonly detected among horseshoe bats with most of the CoVs being found in Chinese horseshoe bats (12, 34, 35). In fact, the two zoonotic *sarbecoviruses*, SARS-CoV and SARS-CoV-2, are both believed to be originated from horseshoe bats. It has been revealed that SARSr-Ra-BatCoV-RaTG13, which is closely related to SARS-CoV-2, was identified from the intermediate horseshoe bat (*Rhinolophus affinis*) (9, 36). A novel sarbecovirus phylogenetically related to SARS-CoV-2 from the little Japanese horse bat (*Rhinolophus cornutus*) was also recently detected in Japan (37). The origin of SARS-CoV was as well discovered to be highly related to bat SARSr-CoV (34, 35, 38). However, it should be noted that a few strains of SARSr-CoVs have been found in Stoliczka’s trident bats (*Aselliscus stoliczkanus*), wrinkle-lipped free-tailed bats (*Chaerephon plicatus*), and intermediate roundleaf bats (*Hipposideros larvatus*) (12, 39, 40). As sarbecoviruses were also detected in other bat species and animals, such as pangolins (family Manidae) and civets (family Viverridae) (41, 42), these animals should also be included in surveillance programs where resources are available. This may help detect new variants of emerging potential and prevent future epidemics.

## Data Availability

The nucleotide sequences of the partial RdRp and S1 fragments of bat-LAMP positive sarbecoviruses have been deposited in the GenBank sequence database under accession no. OR762900 to OR762940.
